# Supplementary material for: The Impact of Non-Invasive Ventilation on Sleep Quality in COPD Patients
Source: J Clin Med. 2022 Sep 19;11(18):5483. doi: 10.3390/jcm11185483 (PMC9504324; doi:10.3390/jcm11185483)
Supplement: Supplementary file 1 [file jcm-11-05483-s001.zip › jcm-1897791-supplementary.pdf]

Journal of Clinical Medicine

MDPI

Online Data Sublement

Table S1. Indication criteria for non-invasive vetiltaion [11]. In addition to the presence of clinical symptoms of chronic respiratory insufficiency, there must be at least one fulfilled criteria.

Diurnal hypercapnia with a $PaCO_2$ >50 mm Hg.
Nocturnal hypercapnia with a $PaCO_2$ >55 mm Hg.
Diurnal hypercapnia with a $PaCO_2$ 46–50 mm Hg and an increase in $PtcCO_2$ >10 mmHg during sleep.
Persistent hypercapnia ($PaCO_2$ >53 mm Hg) at least 14 days after finishing acute ventilation therapy for acute respiratory acidosis.
When decannulation after prolonged weaning is only possible with the help of NIV, even after discharge from hospital.

Table S2. Changes in spirometry, body plethysmography and respiratory muscle function following 3 months of High-intensity non-invasive ventilation (two-tailed t-test for dependent samples)

	**Mean difference*** [confidence interval lower/upper limit]	***P*-value**	***N***
**Spirometry parameters**			
VC [l]	0.2 [0.0/0.4]	**0.026**	25
VC [%predicted]	5.5 [0.3/10.7]	**0.04**	25
FVC [l]	0.1 [-0.0/0.3]	0.120	23
FVC [%predicted]	5.5 [0.7/10.7]	**0.026**	23
FEV_1 [l]	0.1 [0.0/0.1]	**0.038**	25
FEV1 [%predicted]	7.8 [-0.6/16.2]	0.068	25
FEV1/FVC [%]	0.0 [-4.0/4.1]	0.984	23
**Body plethysmography parameters**			
Resistance			
Reff [kPa]	-5.5 [-16.6/5.7]	0.322	25
Reff [%predicted]	-10.9 [-66.8/45.0]	0.691	25
Lung volume			
RV [l]	-0.2 [-0.6/-0.3]	0.498	25
RV [%predicted]	-8.4 [-30.4/13.4]	0.432	25
TLC [l]	0.0 [-0.5/0.5]	0.982	24
TLC [%predicted]	-0.5 [-8.6/7.7]	0.909	24
**Respiratory muscle function**			
P0.1 [kPa]	0.0 [-0.0/0.1]	0.603	20

P0.1 [%predicted]	10.3 [-34.2/54.7]	0.635	20
PImax [kPa]	0.2 [-0.5/0.9]	0.497	20
PImax [%predicted]	4.5 [-4.6/13.5]	0.319	20

Abbreviations: Forced expiratory volume in 1 second= FEV_1; Forced vital capacity= FVC; Residual volume= RV; Total lung capacity= TLC; Body mass index= BMI; Partial pressure of arterialized oxygen= PaO_2; Partial pressure of arterialized carbon dioxide= $PaCO_2$; arterialized standard hydrogen carbonate= HCO_3^-; Pressure during 0.1 sec. inspiratory occlusions= P 0.1; Maximal inspiratory pressure= PI max.

Table S3. Changes in blood gas analysis following 3 months of High-intensity non-invasive ventilation (two-tailed t-test for dependent variables)

	**Mean difference*** [confidence interval lower/upper limit]	***P*-value***	***N***
**Blood gas analysis**			
pH	0.0 [-0.1/0.1]	0.314	14
PO_2	4.0 [-5.6/13.8]	0.192	14
PCO_2	-4.2 [-10.1/1.7]	0.072	14
Transcutaneous PCO_2	-3.9 [-7.7/-0.12]	**0.044**	25

Abbreviations: Partial pressure of arterialized oxygen= PaO_2; Partial pressure of arterialized carbon dioxide= $PaCO_2$.

Figure S1: Boxplot illustrating the results of polysomnographic measurements prior NIV (t1) and following 3 months of noninvasive ventilation (t2) in COPD patients without comorbid obstructive sleep apnea syndrom (n=15).

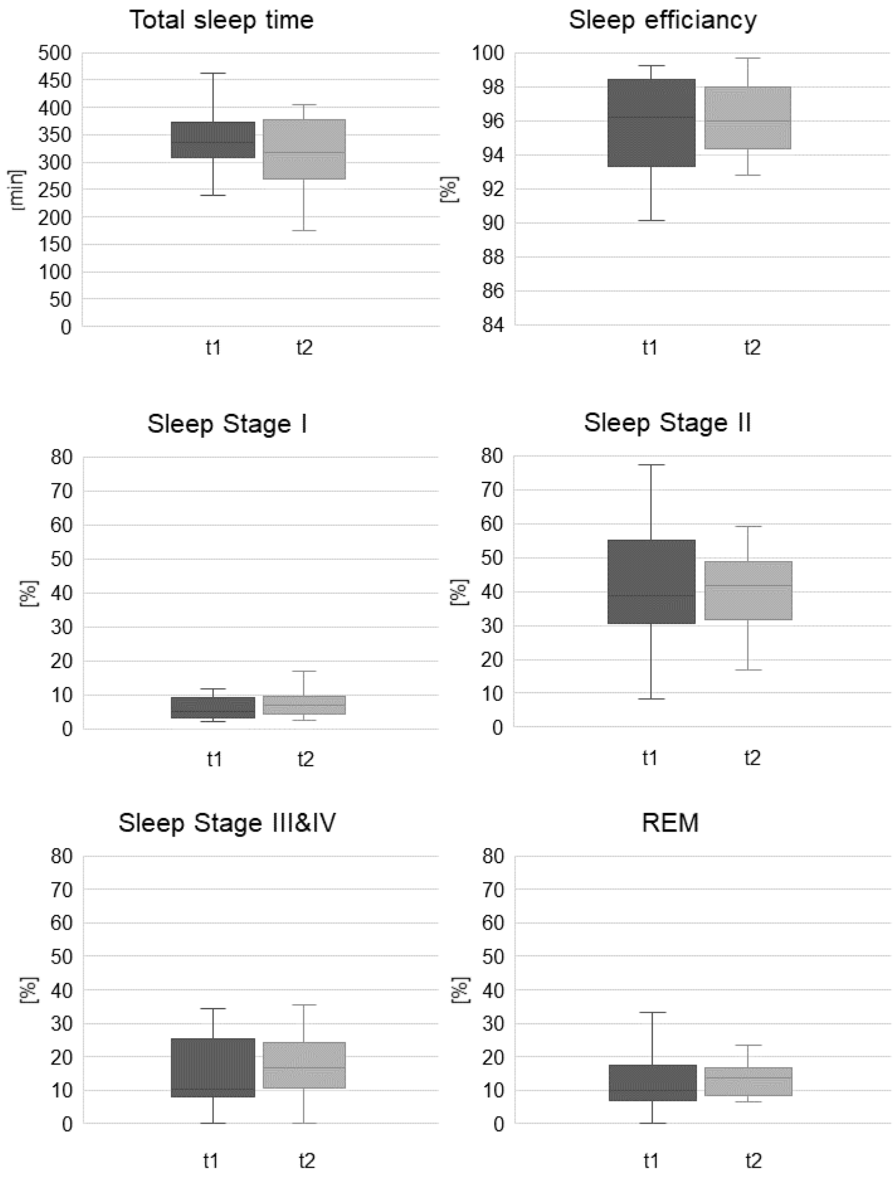

Abbreviations: Minutes= min; Rapid eye movement= REM.

Figure S2: Boxplot illustrating the results of polysomnographic measurements prior NIV (t1) and following 3 months of noninvasive ventilation (t2) in COPD patients with comorbid obstructive sleep apnea syndrom (n=10).

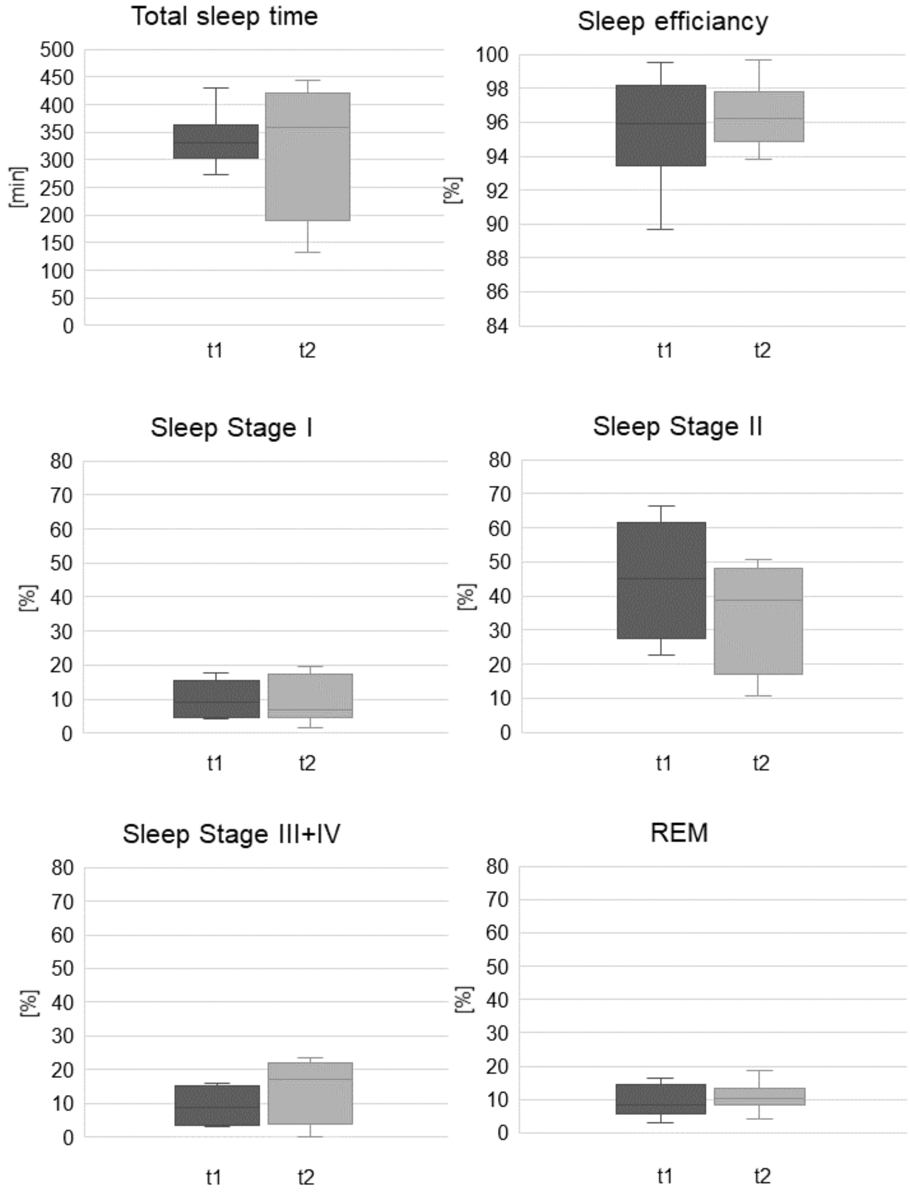

Abbreviations: Minutes= min; Rapid eye movement= REM.
